# Molecular Mechanisms of HIF-1α Modulation Induced by Oxygen Tension and BMP2 in Glioblastoma Derived Cells

**DOI:** 10.1371/journal.pone.0006206

**Published:** 2009-07-09

**Authors:** Francesca Pistollato, Elena Rampazzo, Sara Abbadi, Alessandro Della Puppa, Renato Scienza, Domenico D'Avella, Luca Denaro, Geertruy te Kronnie, David M. Panchision, Giuseppe Basso

**Affiliations:** 1 Hemato-Oncology Laboratory, Department of Pediatrics, University of Padova, Padova, Italy; 2 Department of Neurosurgery, University of Padova, Padova, Italy; 3 Division of Neuroscience and Basic Behavioral Science, National Institute of Mental Health, National Institutes of Health, Bethesda, Maryland, United States of America; Emory University, United States of America

## Abstract

**Background:**

Glioblastoma multiforme (GBM) is one of most common and still poorly treated primary brain tumors. In search for new therapeutic approaches, Bone Morphogenetic Proteins (BMPs) induce astroglial commitment in GBM-derived cells in vitro. However, we recently suggested that hypoxia, which is characteristic of the brain niche where GBM reside, strongly counter-acts BMP effects. It seems apparent that a more complete understanding of the biology of GBM cells is needed, in particular considering the role played by hypoxia as a signaling pathways regulator. HIF-1α is controlled at the transcriptional and translational level by mTOR and, alike BMP, also mTOR pathway modulates glial differentiation in central nervous system (CNS) stem cells.

**Methodology/Principal Findings:**

Here, we investigate the role of mTOR signaling in the regulation of HIF-1α stability in primary GBM-derived cells maintained under hypoxia (2% oxygen). We found that GBM cells, when acutely exposed to high oxygen tension, undergo Akt/mTOR pathway activation and that BMP2 acts in an analogous way. Importantly, repression of Akt/mTOR signaling is maintained by HIF-1α through REDD1 upregulation. On the other hand, BMP2 counter-acts HIF-1α stability by modulating intracellular succinate and by controlling proline hydroxylase 2 (PHD2) protein through inhibition of FKBP38, a PHD2 protein regulator.

**Conclusions/Significance:**

In this study we elucidate the molecular mechanisms by which two pro-differentiating stimuli, BMP2 and acute high oxygen exposure, control HIF-1α stability. We previously reported that both these stimuli, by inducing astroglial differentiation, affect GBM cells growth. We also found differences in high oxygen and BMP2 sensitivity between GBM cells and normal cells that should be further investigated to better define tumor cell biology.

## Introduction

High-grade gliomas and particularly glioblastoma multiforme (GBM), are the most common and most aggressive type of primary brain tumor, accounting for 52% of all primary brain tumor cases. Hypoxia plays a key role in normal homeostasis of stem cells [1] and in the initiation, development and aggressiveness of gliomas, lending support to the concept of a special tumor microenvironment, in which hypoxia could be crucial to recruit cancer stem-like cells, deregulating their differentiation [2], [3]. BMPs treatment has been recently considered a promising therapeutic approach for brain cancer in order to reduce tumor cell growth. Indeed, BMP4 and analogously BMP2 treatment promotes cell cycle arrest and glial differentiation *in vitro* in GBM-derived cells [4]. In a recent work we demonstrated that hypoxia may preserve GBM-derived cells in a more proliferative and less committed cell stage, by down-regulating endogenous BMP signaling in tumor cells and particularly SMAD 1/5/8 activation [3], providing evidence that HIF-1α may counter-act BMP signaling activation under hypoxia. Indication of hypoxia as a regulator of normal and tumor cells growth comes also from other works, in which hypoxia has been shown to induce carotid body growth and generation of new neural crest derived glomus cells [1], while being implicated also in the regulation of several signaling pathways, such as notch signaling [5]. Moreover, HIF-1α expression seems to depend on mammalian target of rapamycin (mTOR) signaling transcriptional and translational control [6]. Furthermore, mTOR signaling pathway seems to be activated also by BMP in murine CNS precursor cells cultured at high density [7]; one of the possible effects mediated by mTOR activation is serine phosphorylation of Stat3, which finally leads to generation of glia [8]. Taken together these data suggest a convergence of BMP with mTOR in controlling glial differentiation and HIF-1α transcriptional activity. Here we investigate the role of mTOR signaling in the regulation of HIF-1α stability in primary GBM-derived cells maintained under hypoxia (2% oxygen), condition resembling their physiological microenvironment [9], evaluating the effects mediated by an acute raise of oxygen tension and by BMP2 treatment. Our results indicate that hypoxia maintains mTOR pathway in an inactive state and this occurs by preserving HIF-1α stability, whereas an acute exposure to high oxygen tension and/or BMP2 treatment promote activation of Akt/mTOR and down stream dependent pro-translational and pro-differentiating responses in GBM cells, which undergo a metabolic shift, as shown by increased of succinate dehydrogenase (SDH) activity following these stimuli. Here we depict the molecular mechanisms occurring in normal and tumor cells after high oxygen tension acute exposure and BMP2 treatment.

## Results

### Acute exposure to high oxygen tension promotes Akt/mTOR activation in a time dependent fashion in GBM-derived cells

**Figure 1:**
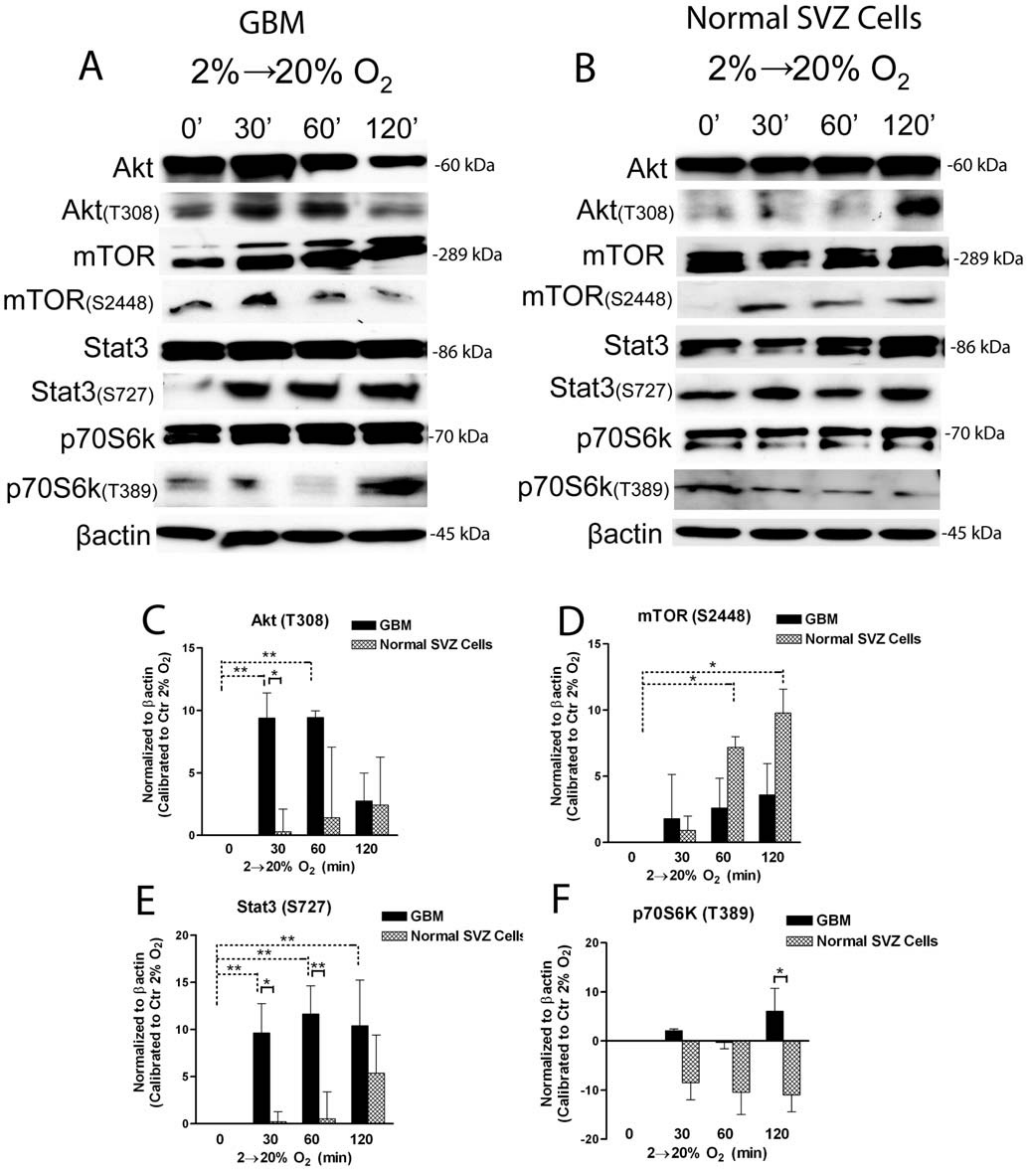
GBM-derived cells undergo Akt/mTOR pathway activation after acute exposure to high oxygen tension. GBM precursors derived cells and normal SVZ derived cells were initially expanded in 2% oxygen, followed by acute exposure to 20% oxygen for 30, 60 or 120 minutes. (A,B) Representative western blot analyses of activated Akt (T308), mTOR (S2448), p70S6K (T389), Stat3 (S727) and total proteins in GBM (A) and normal SVZ cells (B). (C–F) Bar graphs showing mean intensity of indicated proteins normalized to control at 2% oxygen (corresponding to the 0 base line)±S.E.M. comparing 4 different tumors (black bars), n = 3 for each tumor, with 3 different cultures of normal SVZ-derived cells (lighter bars), n = 3 for each culture. Statistical analyses were done comparing each time point for either GBM or normal cells to its respective T0 (0 min in 20% O2), or as indicated by brackets.

It has been previously shown that BMP2 increases Akt serine/threonine kinase activity in serum-deprived 2T3 cells [10], and Akt/PKB signaling is known to activate mTOR pathway. Importantly, we have recently demonstrated that increasing oxygen tension induces activation of endogenous BMP pathway, through SMAD1/5/8 activation, in GBM-derived cells [3]. We sought to investigate if a progressive time dependent exposure of GBM cells, that have been constantly maintained under hypoxia (2% oxygen), to an acute 20% oxygen tension, was promoting Akt/mTOR signaling pathway activation. We observed activation of Akt at the level of threonine 308, but not at the level of serine 473 (data not shown), in a time dependent fashion following high oxygen exposure (Figure 1A,C). Also, mTOR phosphorylation at the level of serine 2448 was induced by high oxygen exposure (Figure 1A,D). Under constant hypoxia these activations were inhibited. mTOR is known to regulate several biological cell responses, amongst them: i) translation, through activation of p70S6-Kinase (p70S6K) and inhibition of the inhibitor 4eBP1, ii) cell proliferation, by acting as a cell cycle regulator, iii) cell survival and cell differentiation, by Stat3 activation, iv) angiogenesis, through activation of HIF-1α and Vascular Endothelial Growth Factor (VEGF). We found that all mTOR downstream targets were activated by acute high oxygen exposure in a time dependent manner. Total Akt/mTOR proteins analyses indicated a homogenous expression among conditions both in GBM and normal cells progressively exposed to high oxygen tension (Figure 1A,B). Particularly, Stat3 (Ser727) was activated after 30 min of high oxygen exposure (Figure 1A,E), this indicating gliogenesis [8] and/or activation of pro-survival response [11]. Moreover, while 4eBP1 was only modestly inhibited by oxygen (data not shown), which may be due to alternative regulatory pathways involved in 4eBP1 regulation, p70S6K (Thr389) was highly up-regulated especially after 120 min (Figure 1A,F). These results indicate that activation of Akt/mTOR dependent pro-translational pathways (p70S6K) and gliogenic and pro-survival mechanisms (Stat3) occur in response to acute high oxygen exposure. Moreover, this increase in translation seems to be directed toward cell differentiation, as indicated by increased p21^cip1^ and increased endogenous BMP dependent astroglial commitment as we reported earlier [3]. In normal SVZ-derived cells Akt was only modestly and not significantly activated by oxygen exposure, while mTOR activation occurred after 1 hr; although mTOR downstream targets Stat3 and p70S6k were not activated following mTOR phosphorylation (Figure 1B–F). These results indicate differences in normal and tumour cell response to high oxygen tension exposure.

**Figure 2:**
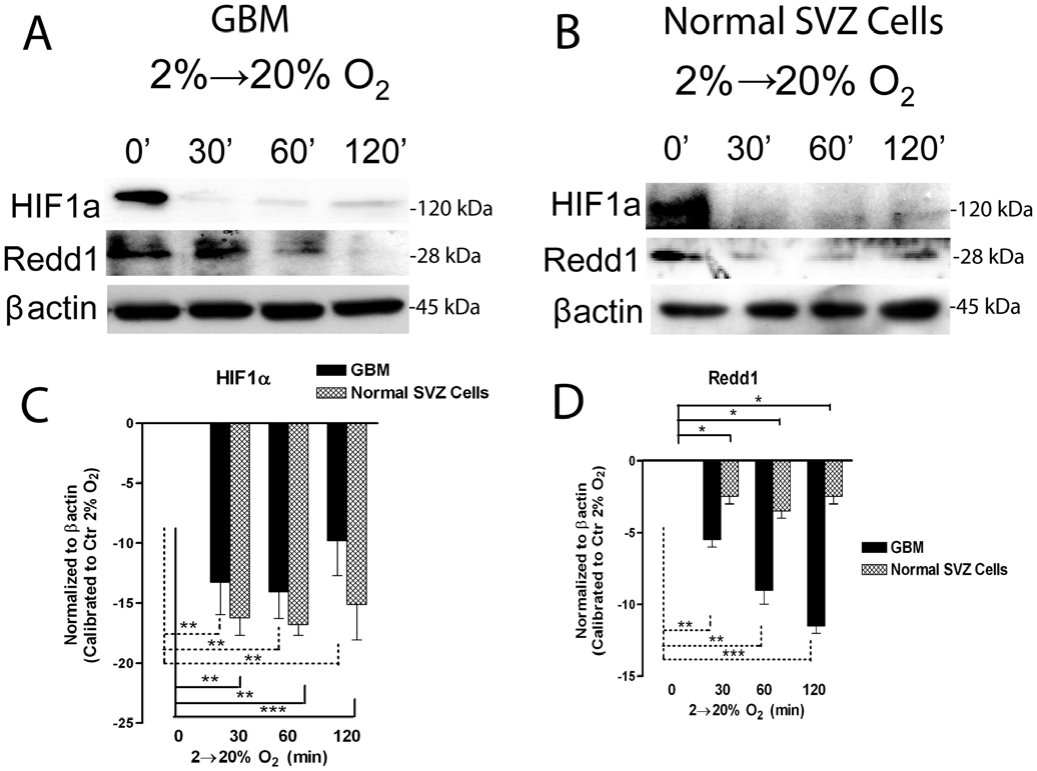
GBM-derived cells undergo transient HIF-1α and REDD1 degradation following acute exposure to high oxygen tension. GBM precursors derived cells and normal SVZ derived cells were initially expanded in 2% oxygen, followed by acute exposure to 20% oxygen for 30, 60 or 120 minutes. (A,B) Representative western blot analyses of HIF-1α and REDD1 in GBM (A) and normal SVZ cells (B). (C,D) Bar graphs showing mean intensity of HIF-1α and REDD1 normalized to control at 2% oxygen (corresponding to the 0 base line)±S.E.M. comparing 6 different tumors (black bars), n = 3 for each tumor, with 3 different cultures of normal SVZ-derived cells (lighter bars), n = 5 for each culture.

Another protein controlled by mTOR is HIF-1α [6]. We found that HIF-1α was rapidly degraded by acute high oxygen exposure, in both GBM and normal SVZ cells (Figure 2A–C). Although, a modest recovery of HIF-1α protein was visible after 2 hr of high oxygen exposure in GBM cells, but not in normal cells (Figure 2A–C), indicating that tumour cells may re-establish HIF-1α level through a hypoxic independent mechanism, possibly controlled by mTOR progressive activation.

We also analyzed REDD1 (RTP801), which has been shown to be strongly induced under hypoxic conditions in a HIF-1α dependent manner [14] and recent studies also suggest that REDD1 plays a role in the TSC1/TSC2-mediated inhibition of mTOR [15]. Accordingly, we found REDD1 downregulated in GBM cells, less intensively in normal SVZ cells, following acute high oxygen exposure (Figure 2A,B,D). Thus, high oxygen dependent Akt/mTOR signaling activation may occur after HIF-1α/REDD1 downregulation.

### HIF-1α is required to repress Akt/mTOR signaling activation in hypoxic tumor cells

**Figure 3:**
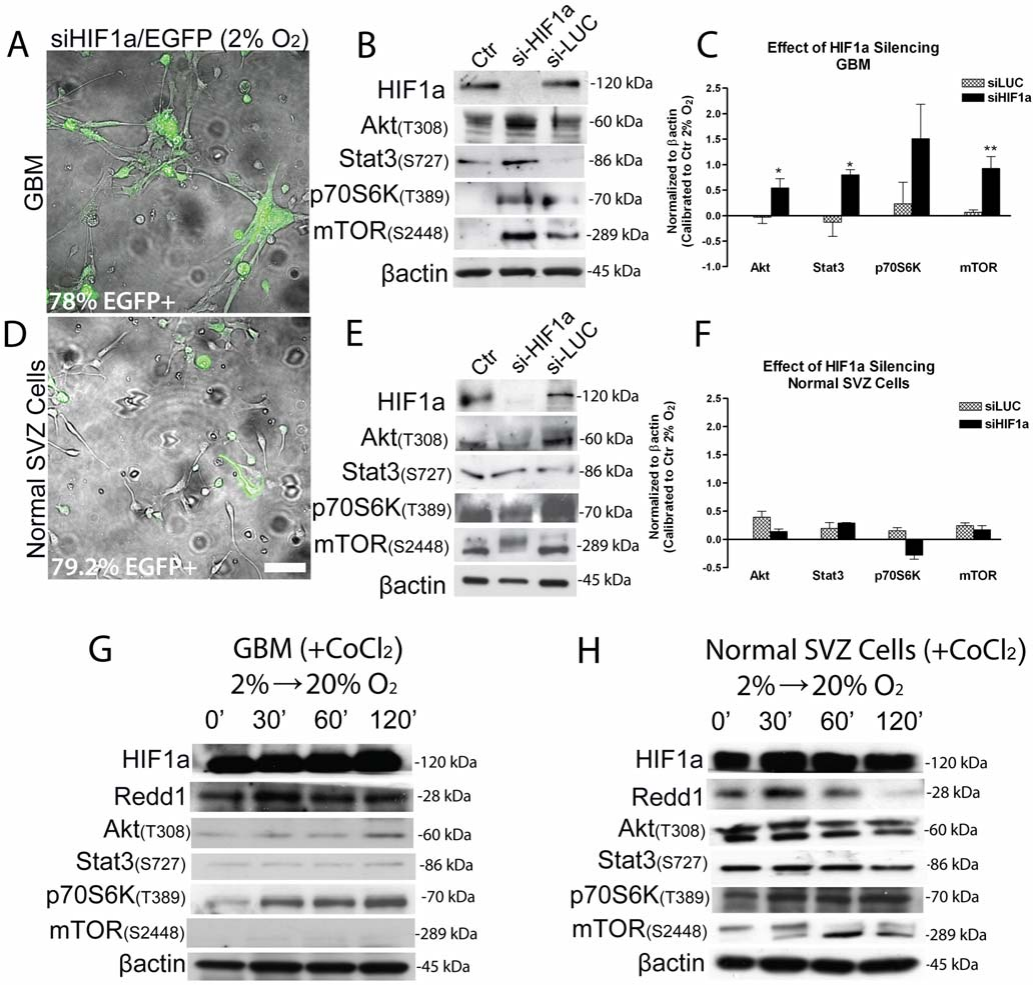
Silencing of HIF-1α promotes Akt/mTOR pathway activation in GBM precursors but not in normal SVZ cells and HIF-1α stabilization by CoCl_2_ maintains Akt/mTOR pathway inhibited following acute high oxygen exposure. (A,D) Representative pictures of GBM (A) and normal SVZ cells (D) transduced using a lentiviral vector containing siHIF-1α along with enhanced green fluorescent protein (siHIF-1α-EGFP) to test the effects of HIF-1α silencing. Same vector containing siLuciferase (siLUC-EGFP) was used to test for non-specific effects (pictures not shown). Percentages of EGFP+ cells was determined by using cytofluorimeter (BD, FC500). (B–E) Representative western blot of HIF-1α silenced GBM cells (B) and normal SVZ cells (E), analyzed for HIF-1α, Akt (T308), mTOR (S2448), p70S6K (T389) and Stat3 (S727), along with β-actin as a loading control. (C,F) Bar graphs showing mean intensity of indicated proteins normalized to control at 2% oxygen (not transduced) (corresponding to the 0 base line)±S.E.M. comparing 2 different tumors (C), n = 2 for each tumor and 1 normal SVZ-derived cell culture (F), n = 2. Asterisks indicate statistically significant differences comparing siHIF-1α to control at 2% oxygen (not transduced). (G,H) Representative western blot analyses of activated Akt (T308), mTOR (S2448), p70S6K (T389), Stat3 (S727) and HIF-1α from GBM (G) and normal SVZ cells (H) that have been pre-incubated 12 hr with CoCl2 (100 µM, Sigma) prior to acute high oxygen exposure. 3 different GBM have been analyzed, n = 2 for each tumor. 20× magnification pictures, bar = 50 µM.

To understand if HIF-1α mediates the repressive effect of low oxygen on Akt/mTOR signaling, we silenced HIF-1α using a lentiviral vector containing siHIF-1α along with enhanced green fluorescent protein as indicator of efficiency of transduction (siHIF-1α-EGFP) (Figure 3A,D), which was compared to a siLuciferase-EGFP vector (siLUC-EGFP) as a negative control [3] (not shown). By silencing HIF-1α in GBM cells a strong differentiation and eventually cell death occurred after 1 week, as already reported in our previous work [3] and these effects were not observed with siLUC-EGFP. Importantly, Akt, mTOR, Stat3 and p70S6K were activated even in HIF-1α silenced cells cultured at 2% oxygen compared to control group and to GBM cells transduced with siLUC-EGFP vector (Figure 3B,C). In normal SVZ cells HIF-1α silencing did not elicit any significant effect and eventually Akt and mTOR inhibition occurred compared to control (Figure 3E,F). HIF-1α silencing correlates with a stronger BMP pathway activity in GBM cells, as shown by SMAD1/5/8 phosphorylation [3], and BMP pathway is known to activate Akt [10] and to be correlated to Stat3 regulation in promoting astrogliogenesis [12]. These results indicate that HIF-1α in hypoxic tumor cells may be required to repress convergent signals (SMAD1/5/8 and Stat3) directed to promote an astroglial fate. To prove that HIF-1α downregulation is required in evoking high oxygen dependent Akt/mTOR activation we stabilized HIF-1α by using cobalt chloride (CoCl_2_, 100 µM), which mimics the effect of hypoxia on HIF-1α, and 12 hr later we exposed cells to high oxygen tension. Performing these experiments directly on HIF-1α silenced cells was not feasible given the scarce viability following HIF-1α silencing. We found that by chemically stabilizing HIF-1α, REDD1 was upregulated and Akt/mTOR pathway was maintained inhibited in GBM derived cells exposed to high oxygen tension (Figure 3G), only p70S6K activation did occur and this may depend on effectors of p70S6K activation alternative to mTOR. Importantly, normal SVZ cells responded in a different way; indeed, by chemically stabilizing HIF-1α, REDD1 was transiently upregulated and Akt/mTOR pathway was not inhibited, unlike in tumor cells, following exposure to high oxygen tension (Figure 3H), and Akt and Stat3 were eventually upregulated by CoCl_2_. These results indicate that HIF-1α stabilization, probably through REDD1, prevents high oxygen induced Akt/mTOR activation, and this seems to occur more specifically in GBM derived cells.

### Exogenous BMP2, analogously to high oxygen exposure, promotes Akt/mTOR pathway activation and this depends on HIF-1α/REDD1 downregulation

**Figure 4:**
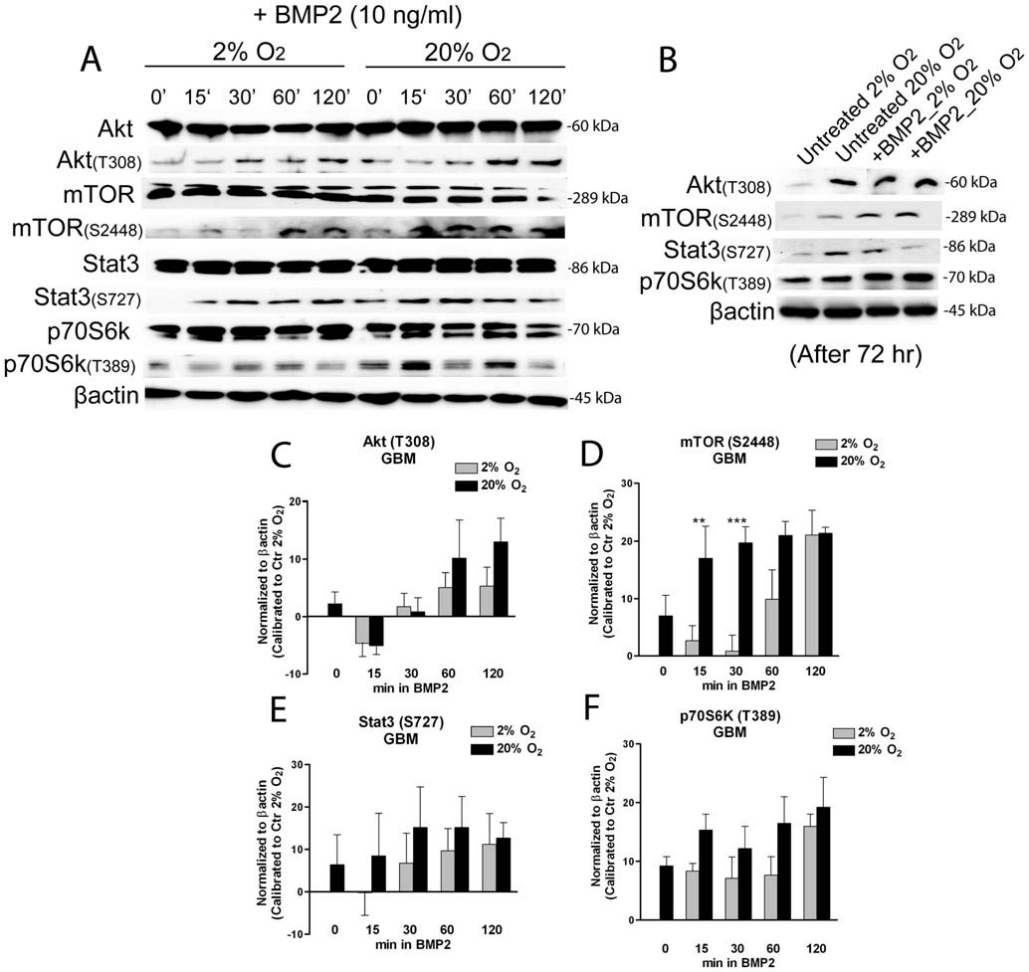
Exogenous BMP2 promotes Akt/mTOR activation in GBM-derived cells and these effects are reduced by hypoxia. (A,B) Representative western blot analyses of activated Akt (T308), mTOR (S2448), p70S6K (T389), Stat3 (S727) and total proteins; GBM-derived cells, initially expanded in 2% oxygen were acutely exposed to 50 ng/ml BMP2 for 0, 15, 30, 60 or 120 minutes, either maintained at 2% or transferred at 20% oxygen (A). Also, some GBM-derived cells were treated with 10 ng/ml BMP2 for longer time (72 hrs) (B). (C–F) Bar graphs showing mean intensity of indicated proteins normalized to control at 2% oxygen (corresponding to the 0 base line)±S.E.M. comparing 5 different tumors, n = 3 for each tumor.

We sought to investigate if exogenous BMP2, alike acute high oxygen exposure, could affect Akt/mTOR pathway activation. While total Akt/mTOR signaling proteins resulted to be homogenously expressed both in GBM and normal cells after BMP2 treatment (Figure 4A and Figure S1A), we found that activation of Akt (Thr308) and also mTOR (Ser2448) occurred with time (Figure 4A,C,D), and, importantly, this activation was accelerated and improved under acute high oxygen exposure, remaining highly activated even after a long term BMP2 exposure (72 hr) (Figure 4B). Importantly, under hypoxia Akt and mTOR were inhibited after short time treatment, resulting activated after 72 hr of BMP2 stimulus. In normal SVZ cells we found that Akt and mTOR were only transiently modulated by BMP2 addition (Figure S1A,C,D) and after 72 hr these activations resulted down-regulated (data not shown). Analyses of mTOR downstream targets, revealed a time dependent increase of Stat3 (Ser727) and p70S6K (Thr389) activation following BMP2 treatment, and these effects were decelerated and decreased by maintaining cells under hypoxia (Figure 4A,E,F), and this occurred transiently also in normal cells (Figure S1A,E,F). Also p21^cip1^, involved in cell cycle arrest and induction of differentiation, was found up-regulated by BMP2, but hypoxia inhibited this effects (data not shown).

**Figure 5:**
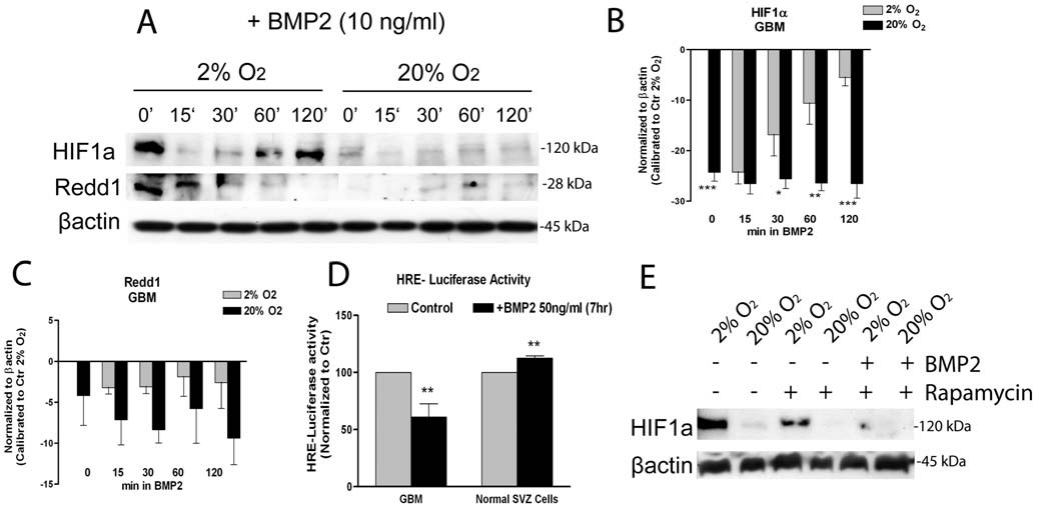
Exogenous BMP2 controls HIF-1α protein level and HIF-1α dependent transcriptional activity even under hypoxia. (A) Representative western blot analyses of HIF-1α and REDD1; GBM-derived cells, initially expanded in 2% oxygen were treated as described in Fig. 4. (B,C) Bar graphs showing mean intensity of HIF-1α and REDD1 normalized to control at 2% oxygen (corresponding to the 0 base line)±S.E.M. comparing 6 different tumors, n = 3 for each tumor. (D) HRE-luciferase assay: GBM cells and normal SVZ-derived cells were transfected either with a HRE-firefly luciferase reporter construct or with a mutated HRE version of the same construct to evaluate aspecific effects. Along with these vectors, also a Renilla luciferase vector has been transfected in order to normalize luciferase detection. Normalization of the data to the mutated HRE vector was done and then values were calibrated to untreated cells (Control). 3 different GBM have been analyzed, n = 2 for each tumor. (E) Representative western blot analysis of HIF-1α in GBM-derived cells, initially expanded in 2% oxygen, then acutely exposed to 100 µM rapamycin alone or in combination with 10 ng/ml BMP2 for 72 hrs. β-actin was used as a loading control. 2 different GBM have been analyzed, n = 3 for each tumor.

HIF-1α expression is known to be controlled by mTOR [6] and REDD1, mTOR inhibitor, is transcriptionally activated by HIF-1α [14]. We analyzed if HIF-1α protein was affected following a progressive BMP2 treatment and we found that HIF-1α was strongly down-regulated even under hypoxia following 15 min of BMP2 stimulation in GBM cells (Figure 5A,B). Importantly, HIF-1α level was recovered after 120 min, however not when GBM cells were also acutely exposed to high oxygen. This later stabilization may be consequential to the BMP2 mediated mTOR signaling activation (Figure 4A,B). According to our recent work [3], HIF-1α level was reduced after a longer BMP2 exposure (72 hrs) (not shown), which is likely correlated to BMP2 dependent glial differentiation in GBM cells. Oppositely, maintenance of HIF-1α expression seems to correlate to tumour cells de-differentiation and normal cells primitiveness [5], [13]. In normal SVZ-derived cells HIF-1α was transiently down-regulated following BMP2 stimulation and with time it was recovered (Figure S1A,G), but its expression did not change after long term (72 hr) treatment (data not shown). Importantly, also REDD1 was down-regulated by BMP2 under hypoxia (Figure 5A,C), and this was only transiently occurring in normal cells (Figure S1A). This suggests that BMP2, analogously to an acute high oxygen exposure, may promote mTOR activation by down-modulating inhibitory REDD1.

As REDD1 is transcriptionally activated by HIF-1α [14], we investigated if BMP2 besides modulating HIF-1α protein, promoted also inhibition of HIF-1α transcriptional activity, by using a hypoxia responsive element (HRE)-luciferase reporter construct. Despite a physiologic tumour samples variability, we recorded a 40% reduction of HIF-1α transcriptional activity under hypoxia following 8 hr of BMP2 treatment compared to untreated cells. Conversely, in normal SVZ cells BMP2 was operating in an opposite way, promoting a nearly 10% increase of HIF-1α activity (Figure 5D), pointing to differences between normal and tumor cells in BMP2 responsiveness.

We also tested if rapamycin treatment alone or in combination with BMP2 to evaluate if blocking mTOR downstream signaling affected also HIF-1α stability. We found that 72 hr of rapamycin treatment, which did not affect HIF-1α transcriptional activition (data not shown), induced a less pronounced HIF-1α protein reduction, in accordance with reported results [16], whereas in combination with BMP2 these effects were not further improved (Figure 5E). Normal SVZ cells responded in a similar fashion (data not shown).

**Figure 6:**
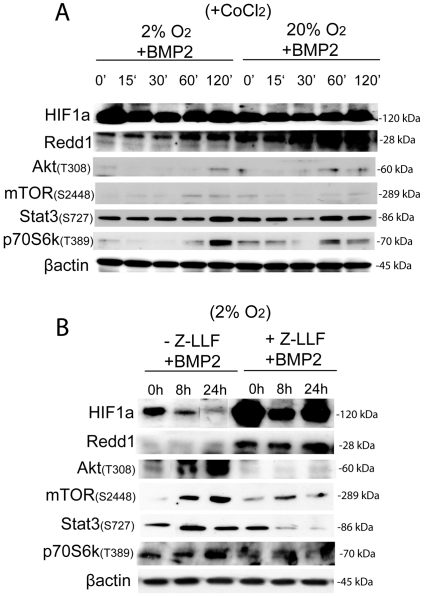
HIF-1α stabilization and consequential REDD1 upregulation maintains Akt/mTOR signaling inhibited in BMP2 treated GBM derived cells. (A) Representative western blot analyses of HIF-1α, REDD1, activated Akt (T308), mTOR (S2448), p70S6K (T389), Stat3 (S727), along with β-actin as loading control; GBM cells have been treated for 12 hr with CoCl_2_ (100 µM, Sigma) either at 2% oxygen or 20% oxygen, starting progressive time course treatment with BMP2 the day after. 2 different GBM have been analyzed, n = 1 for each tumor. (B) Representative western blot analyses of indicated proteins extracted from GBM derived cells that have been pre-treated with Z-LLF-CHO, proteasome inhibitor, added 30 min prior to BMP2 treatment (for 8 or 24 hr). 2 different GBM have been analyzed, n = 1 for each tumor.

To prove that Akt/mTOR activation was due to inhibition of HIF-1α/REDD1 by BMP2, we performed BMP2 treatment on CoCl_2_ pretreated GBM cells. We found that by stabilizing HIF-1α and consequentially also REDD1, Akt/mTOR signaling was not activated following BMP2 treatment (Figure 6A). p70S6K activation lately occurred, probably due to effectors of p70S6K activation alternative to mTOR, as commented above, and Stat3 (S727) was eventually more stably expressed in CoCl_2_ treated cells, although it was not modulated by BMP2 during time (Figure 6A). Importantly, in normal SVZ cells, chemically HIF-1α stabilization was only transiently inducing REDD1 upregulation and Akt/mTOR pathway was not inhibited, unlike in tumor cells, following BMP2 treatment (Figure S1B), and Akt and Stat3 were eventually upregulated. Alternatively to the use of CoCl_2_, we used the proteasome inhibitor Z-LLF-CHO (Z-LLF, 30 µM) added 30 min prior to 8 or 24 hr of BMP2 time treatment under hypoxia. By preventing proteasomal degradation, BMP2 did not affect HIF-1α protein stability (Figure 6B). Moreover, REDD1 was upregulated and Akt/mTOR signaling was not activated following BMP2 treatment under hypoxia (Figure 6B). These results confirm that HIF-1α degradation and consequentially also REDD1 downregulation are required in BMP2 dependent Akt/mTOR activation, and this preferentially occurs in GBM cells.

### Exogenous BMP2 promotes increase of PHD2 protein level even under hypoxia by dowregulating FKBP38

**Figure 7:**
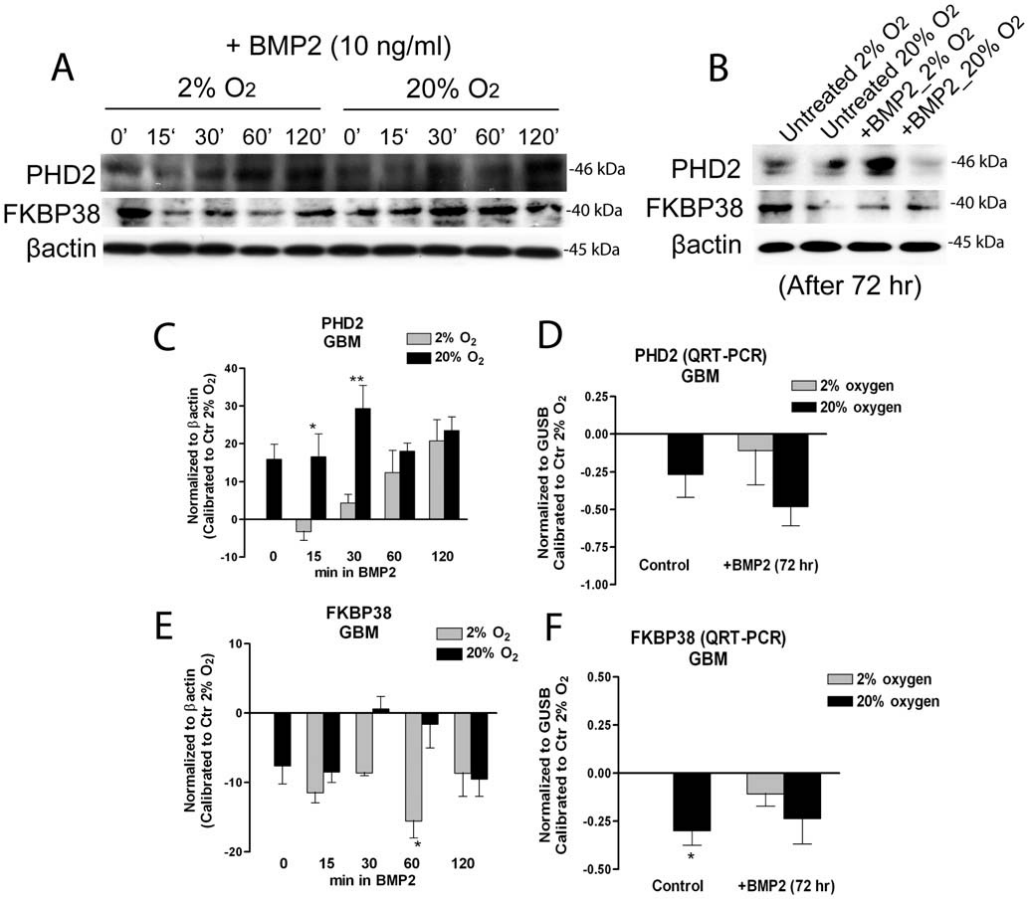
Exogenous BMP2 promotes increase of PHD2 protein level even under hypoxia by dowregulating FKBP38. (A,B) Representative western blot analyses of PHD2 and FKBP38; GBM-derived cells, initially expanded in 2% oxygen were treated as described in Fig. 4. Also, some GBM-derived cells were treated with 10 ng/ml BMP2 for longer time (72 hrs) (B). (C,E) Bar graphs showing mean intensity of PHD2 and FKBP38 proteins normalized to control at 2% oxygen (corresponding to the 0 base line)±S.E.M. comparing 6 different tumors, n = 3 for each tumor. (D,F) QRT-PCR analyses of *PHD2* and *FKBP38*, normalized to *GUSB* and then calibrated to 2% oxygen control (ΔΔCt Method), mean±S.E.M. comparing 3 different GBM, n = 3 for each tumor.

As we saw that HIF-1α protein stability was affected following BMP2 treatment, we investigated if proline hydroxylases (PHDs), involved in HIF-1α proline hydroxylation and consequential proteasomal degradation, were modulated by BMP2. PHD2 in particular has been described as the critical oxygen sensor setting the low steady-state levels of HIF-1α in normoxia [17]. In the same work PHD2 was found up-regulated by hypoxia, providing a HIF-1-dependent auto-regulatory mechanism driven by oxygen tension itself. We found that PHD2 protein was rapidly up-regulated by BMP2, and this occurred more slowly under hypoxia, in both GBM (Figure 7A,C) and normal SVZ cells (Figure S1A,H). Also after a prolonged BMP2 treatment PHD2 protein resulted up-regulated in GBM cells (Figure 7B), which is in accordance to our previous work [3]. QRT-PCR revealed that while *PHD2* transcript was up-regulated under hypoxia, according to literature [17], 72 hr of BMP2 treatment induced a modest but not significant *PHD2* mRNA decrease (Figure 7D). *PHD2* transcript reduction was recorded also in BMP2 treated normal SVZ cells (Figure S1I). Notably, PHD2 promoter is characterized by the presence of HRE consensus sequences directly controlled by HIF-1α [18]. Thus, we hypothesize that the observed *PHD2* mRNA reduction may depend on HIF-1α transcriptional inhibition induced by BMP2, as previously described (Figure 5D).

Following-up on our observation that BMP2 mediated PHD2 protein increase was not dependent on de novo protein synthesis (Figure 7D), we sought to investigate if BMP2 was somehow increasing PHD2 protein stability. Peptidyl prolyl cis/trans isomerase FK506-binding protein 38 (FKBP38) has been described as a PHD2 protein regulator by targeting PHD2 to proteasome degradation [19]. We found that FKBP38 was progressively down-regulated by BMP2 under hypoxia (Figure 7A,E), and this effect was observed also after a prolonged BMP2 treatment (Figure 7B). Conversely to what has been described [19], we also found that FKBP38 was up-regulated in hypoxic GBM cells, suggesting that it may be involved in hypoxic HIF-1α stability by promoting PHD2 degradation. QRT-PCR analysis confirmed that *FKBP38* was down-regulated by acute exposure to high oxygen and by BMP2 under hypoxia also at the transcriptional level (Figure 7F). Opposite, this was not occurring in normal SVZ-derived cells in which BMP2 treatment combined with high oxygen exposure was inducing *FKBP38* up-regulation (Figure S1J), also at the protein level (Figure S1A,K). Thus, we hypothesize that BMP2 may promote PHD2 stabilization by down-modulating FKBP38 expression in GBM cells.

### Exogenous BMP2, by decreasing intracellular succinate, increases PHD2 activity leading to HIF-1α modulation

**Figure 8:**
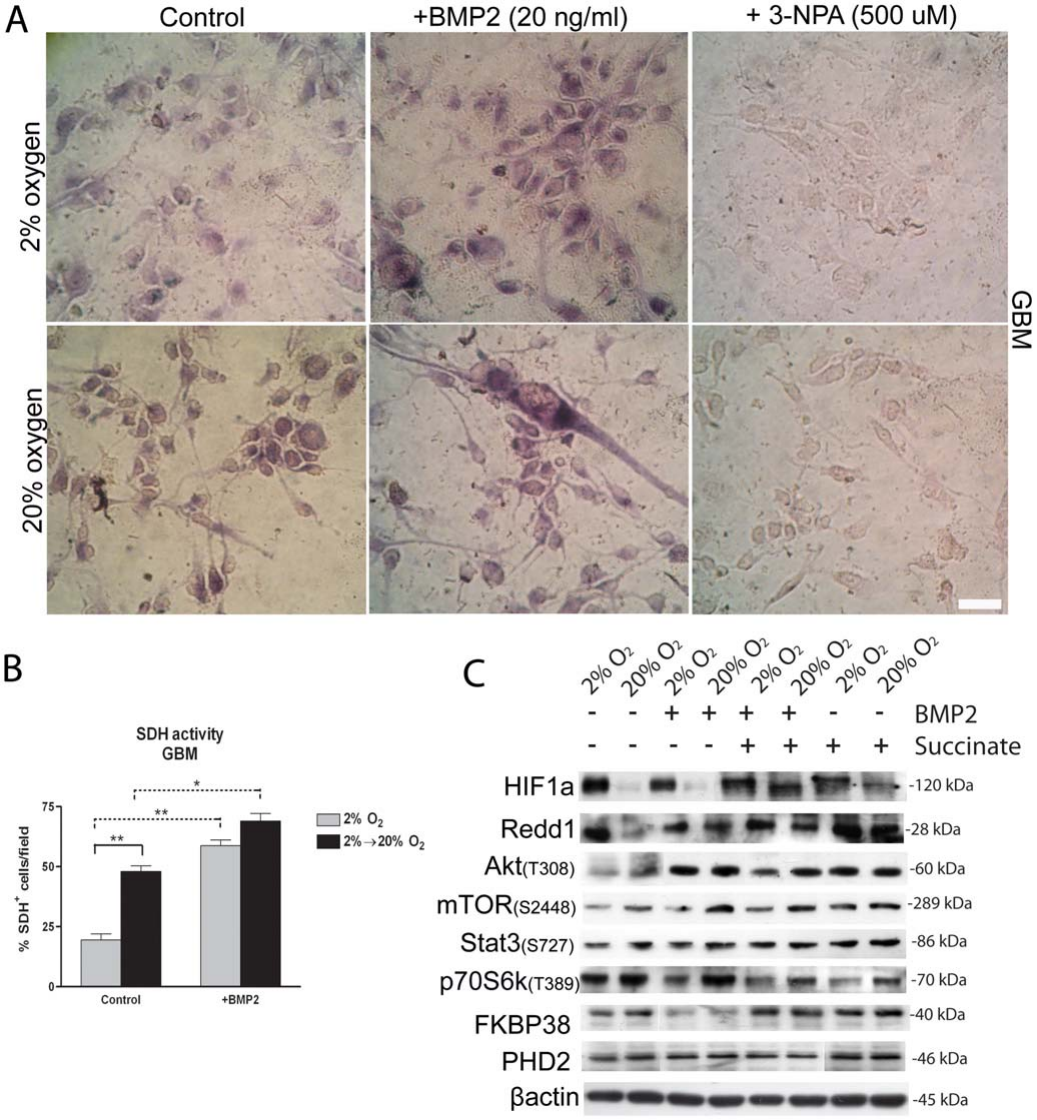
Exogenous BMP2 promotes higher SDH activity and addition of exogenous succinate upregulates HIF-1α/REDD1 and FKBP38 proteins in BMP2 treated cells. (A) Representative citochemical analysis of SDH activity by using NBT reduction methodology. Cells were incubated for 48 hr in presence of either BMP2 (30 ng/ml) or 3-nitropropionic acid (3-NPA) (500 µM, Sigma), known to irreversibly inactivate SDH (negative control). (B) Bar graph showing mean percentage of SDH^+^ cells (blue-violet cells) counted from 40× magnification pictures (picture area = 0.02 cm^2^), bar = 20 µM. 2 different tumors have been used, n = 2 for each tumor. (C) Representative western blot analyses of indicated proteins extracted from cells that have been treated 72 hr in presence of either BMP2 alone (10 ng/ml), or diethyl-succinate alone (5 mM, Sigma) or combined BMP2 and diethyl-succinate. 2 different GBM have been analyzed, n = 2 for each tumor.

We finally sought to investigate if BMP2 treatment increased PHD2 activity thus causing HIF-1α modulation. It has been previously reported that impaired SDH activity in several cancer types is associated to HIF-1α stabilization, through a mechanism involving intra-cytoplasmic succinate accumulation and consequential PHD2 inhibition [20]. Indeed, SDH is the enzyme complex II bound to the inner mitochondrial membrane that converts succinate to fumarate via FAD reduction to FADH2. Importantly, it has also been shown in other cell models that induction of differentiation by BMPs increases mitochondrial oxidative phosphorylation, as seen by higher SDH activity [21]. We found that BMP2 treatment induced increase of SDH activity in GBM cells (Figure 8A,B). This suggests that pro-differentiating agents, such as BMPs, may promote a metabolic shift toward oxidative phosphorylation in tumor cells and that BMP2, by decreasing intracellular succinate through induction of SHD activation, may increase PHD2 activity leading to HIF-1α modulation. Notably, in normal SVZ cells SDH activation was not changed following BMP2 treatment (Figure S2A), this indicating differences in metabolic response to BMP2 treatment between normal and tumor cells. Further addition of exogenous esterificated diethyl-succinate (5 mM, Sigma) either in presence or absence of BMP2 did not induce SDH activation (not shown).

We also tested if additional succinate in combination with BMP2, was promoting a recovery of HIF-1α protein level and/or determining an inhibition of Akt/mTOR signaling in GBM cells. We found that HIF-1α, REDD1 and FKBP38 proteins were upregulated in BMP2/succinate treated cells, even when exposed to 20% oxygen. Moreover, in particular Akt and p70S6K activation were modestly decreased in presence of combined BMP2 and succinate, as compared to BMP2 only treated cells (Figure 8C). This indicates that additional succinate, by upregulating HIF-1α and REDD1, partially inhibits Akt/mTOR activation following BMP2 treatment.

## Discussion

HIF-1α and hypoxia have been shown to be implicated in tumour progression [22]–[27]. We have recently shown [3] that BMP2 in vitro treatment, known to promote glial differentiation in GBM derived cells [4], resulted to be less effective under hypoxia, suggesting that hypoxia and also HIF-1α may preserve GBM tumour cell stemness by de-sensitizing cells to pro-differentiating BMP2 stimulus [3]. It has also been reported that epigenetic-mediated dysfunction of the BMP receptor-IB (BMPR-IB) inhibits differentiation of glioblastoma-initiating cells [28], but the role of hypoxia in BMP pathway regulation has not been previously considered in other studies. Moreover, HIF-1α expression seems to depend on mTOR signaling control [6] and mTOR seems to be activated also by BMP in murine CNS precursor cells cultured at high density [7]. These observations suggest a convergence of BMP with mTOR in controlling glial differentiation and on the other hand HIF-1α transcriptional activity. In this study we investigated the role of mTOR signaling in the regulation of HIF-1α stability in primary GBM-derived cells compared to normal SVZ-derived cells, maintained under hypoxia, evaluating the effects mediated by acute high oxygen exposure and BMP2. We found that acute exposure to high 20% oxygen tension promotes Akt and mTOR signaling pathways activation, with consequential increase of mTOR downstream targets (Stat3 and p70S6K), whereas hypoxia inhibits these effects. Importantly, under hypoxia Akt/mTOR pathway results to be inhibited. p70S6K leads to activation of pro-translational responses, which dependably leads to increased differentiation. Accordingly, we also found Stat3, known to promote astrogliogenesis [8], highly activated by acute high oxygen tension. In a previous study it has been reported that reactivation of Stat3 in PTEN-deficient glioblastoma cells inhibits their proliferation and invasiveness [29]. We previously found that after acute high oxygen exposure also SMAD1/5/8 activation, known to induce astrogliogenesis, occurred [3], analogously to Stat3 activation. Notably, normal SVZ-derived cells underwent mTOR but not analogous Akt, Stat3 and p70S6K activation following high oxygen acute exposure, suggesting differences in oxygen sensitivity between tumour and normal cells. Importantly, after high oxygen exposure HIF-1α was down-modulated, but after 120 min it was partially reconstituted in GBM probably by a mTOR dependent mechanism. Indeed, HIF-1α has been found to be regulated by mTOR via a mTOR signaling motif, this leading to increased angiogenesis [6]. Recent studies also indicate that REDD1 (RTP801), induced under hypoxic conditions in a HIF-1α dependent manner [14], plays a role in the TSC1 (hamartin)/TSC2 (tuberin)-mediated inhibition of mTOR [15], these indicating a reciprocal regulatory control between HIF-1α and mTOR. Here we found that by stabilizing HIF-1α with CoCl_2_, inhibition mTOR pathway was maintained following acute high oxygen exposure on tumor cells, and this may occur through a negative feedback loop dependent on HIF-1α mediated transcriptional activation of REDD1. Accordingly, we found that silencing of HIF-1α promotes stronger activation of Akt/mTOR even at 2% oxygen, analogously to inducing SMAD1/5/8 activity [3]. Together these results indicate that HIF-1α may be required to repress besides SMAD1/5/8, also Akt and Stat3 activation in hypoxic GBM cells, molecular signals directed toward induction of astroglial fate in neural stem cells [30].

It has been shown that BMP2 increases Akt serine/threonine kinase activity in serum-deprived 2T3 osteoblasts [10] and accordingly we found that exogenous BMP2 treatment, analogously to acute high oxygen exposure, induced up-regulation of Akt/mTOR pathway, particularly of Stat3. Importantly, these effects were downregulated both by hypoxia and by HIF-1α, whose stabilization obtained with either CoCl_2_ or by using proteasome inhibitor (Z-LLF) was preventing BMP2 induced Akt/mTOR activation, especially in tumor cells. In normal SVZ cells Akt, mTOR and its downstream targets were only transiently modulated by BMP2, indicating a different sensitivity between tumor and normal cells in responsiveness to exogenous BMP2.

Importantly, we also found that after 15 min of BMP2 treatment a rapid down-regulation of HIF-1α protein occurred under hypoxia, but its level was recovered during time in GBM cells. After a longer BMP2 treatment (72 hr), when glial commitment is at a more advanced stage, we previously described [3] a reduction of HIF-1α protein, regardless the presence of hypoxia, unlike in normal SVZ cells. We also found that following BMP2 treatment REDD1, HIF-1α downstream target [14] and mTOR inhibitor [15], was down-regulated even under hypoxia. Thus, BMP2, analogously to an acute exposure to high oxygen tension may promote mTOR activation by down-modulating REDD1, as a consequence of HIF-1α dependent transcriptional activity inhibition (Figure 5D).

PHD2 is involved in hydroxylation and consequentially degradation of HIF-1α; we found that BMP2 induced increase of PHD2 protein level, in both normal and tumour cells, but this upregulation does not seem to be related to increased protein translation. Moreover, FKBP38, which has been recently described as a PHD2 protein modulator [19], was down-regulated both by high oxygen exposure and BMP2 treatment in GBM cells maintained under hypoxia. FKBP38 has been described to bind to mTOR, inhibiting the ability of mTORC1 to signal to downstream targets [31]. However, the molecular functions of FKBP38 remain still elusive. We hypothesize that a decrease of FKBP38 following BMP2 and high oxygen exposure may stabilize PHD2 protein level leading to HIF-1α degradation even under hypoxia. FKBP38 downregulation may also explain mTOR signaling activation under these stimuli.

Since several decades it has been shown that solid tumor cells are characterized by intense anaerobic glycolysis [32], strongly suggestive of an association between mitochondrial dysfunction and cancer. Indeed, a variety of tumor cell types are characterized by an impaired respiratory capacity [32], and this was confirmed also in our in vitro culture conditions, as shown by increased lactic acid production and decreased cytochrome c oxidase activity (COX) in GBM cells compared to normal SVZ cells (Figure S2B,C). It has also been shown that mTOR pathway is influenced by the intracellular concentration of ATP, independent from the abundance of amino acids [33]. Thus, we hypothesize that mTOR signaling down-regulation in our hypoxic GBM cells may be due also to decreased ATP availability.

Among mitochondrial dysfunctions, impaired SDH activity in several cancer types has been associated to non-hypoxic HIF-1α stabilization, through a mechanism involving intracytoplasmic succinate accumulation and consequential PHD2 inhibition [20]. Importantly, it has also been shown in other cell models that induction of differentiation by BMPs increases mitochondrial oxidative phosphorylation, as seen by higher SDH activity [21]. Although, this effect has not been clearly described in tumor cells. We found that SDH activity was increased following BMP2 treatment, suggesting that pro-differentiating agents, such as BMPs, may promote a metabolic shift toward oxidative phosphorylation also in tumor cells. Addition of succinate in combination with BMP2, was promoting a recovery of HIF-1α protein, upregulation of REDD1 and consequentially a moderate inhibition of Akt/mTOR signaling, especially of p70S6K.

**Figure 9:**
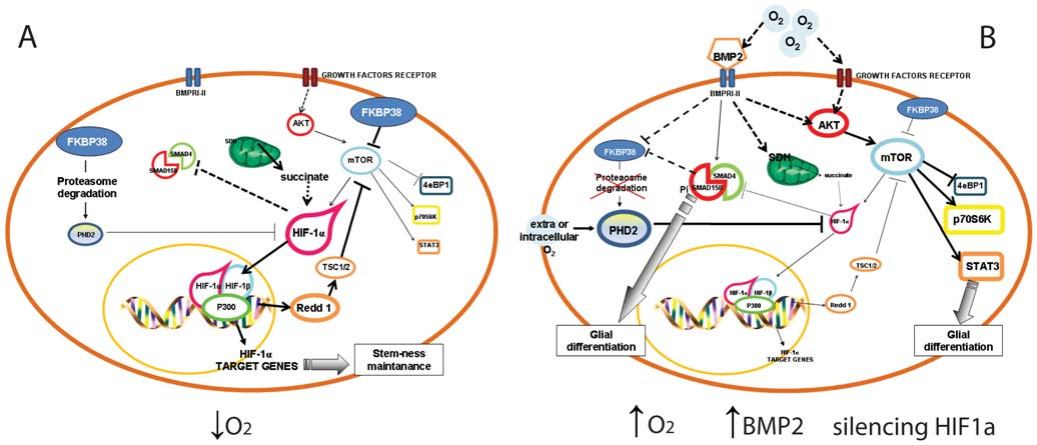
Summary of the hypothetical GBM cell responses to the niche. (A) Under hypoxia, brain tumor cells undergo HIF-1α stabilization, due to PHD2 degradation probably mediated by FKBP38. Consequentially, HIF-1α dependent transcriptional control occurs, with inhibition of Akt/mTOR signaling probably through REDD1 and TSC1/2. Moreover, HIF-1α inhibits SMAD1/5/8 activation [3]. Under hypoxia tumor cells remain in an undifferentiated cell state [2]. (B) Raise of extracellular/intracellular oxygen tension and/or exogenous BMP2, or silencing of HIF-1α, besides activating BMP signaling [3], promote activation of Akt/mTOR pathway. Consequentially mTOR induces activation of its downstream targets, such as Stat3 (pro-survival, glial differentiation) and p70S6K (pro-translational responses). Under these conditions, HIF-1α, which is also controlled by mTOR at the transcriptional and translational level, is down-regulated especially in the long term [3]. We showed that HIF-1α degradation is also related to BMP2 dependent increase of SDH activity, this leading to intracellular succinate level decrease and consequential PHD2 activation, in accordance with previous report [20]. We hypothesize that BMP signaling, either independent or dependent on SMAD1/5/8 activation, may also down-regulate FKBP38, thus impeding PHD2 proteasomal degradation and consequentially stabilizing PHD2 protein. Moreover, BMP2 dependent FKBP38 downregulation correlates also to activation of mTOR signaling, as FKBP38 is known to be a mTORC1 inhibitor [31].

In conclusion, we describe the mechanisms by which BMP2 and, analogously, oxygen tension perturbation, by activating Akt/mTOR signaling, inhibiting FKBP38 and by inducing SDH activity modulate HIF-1α stability and consequentially REDD1 transcription in GBM cells. Together these effects are restrained by preserving the hypoxic niche (Figure 9). Moreover, our results point to discrete differences in high oxygen and BMP2 sensitivity between GBM cells and normal cells that should be exploited to better define tumor cell biology.

## Materials and Methods

### Isolation and gas-controlled expansion of cells

Normal precursor cells were derived from brain subventricular zone (SVZ) tissue of 3 premature neonates who died shortly after birth (HuSC23, HuSC27 and HuSC30), previously characterized [34] and hereafter termed normal SVZ precursors (see Table 1).

**Table 1:** Brain Tumors and Normal Neural Progenitor Cells used in study.

Code	Tumor Type	Age	Gender
**HuTuP01**	Glioblastoma	65	male
**HuTuP02**	Glioblastoma	43	female
**HuTuP10**	Glioblastoma	75	female
**HuTuP11**	Glioblastoma	52	female
**HuTuP13**	Glioblastoma	67	male
**HuTuP15**	Glioblastoma	76	female
**HuTuP47**	Glioblastoma	81	female
**Post-mortem review of non-tumour tissue source**			
**HuSC23**	Premature polycythemic twin (of a twin-to-twin transfusion) who died on the day after birth	23 gw	male
**HuSC27**	Premature infant who died of pulmonary complications 2 weeks after birth	23 gw	male
**HuSC30**	Premature fraternal twin who died on the day after birth, product of in vitro fertilization	25 gw	male

Brain tumours were acquired directly from surgery, dissociated and cells were expanded in culture. First neuropathological review of the tumour tissue was followed by a second independent review. Patient ages listed in years (y). Non tumour tissue was from premature infants, listed in gestational weeks (gw), who died shortly after birth; periventricular zone tissue from the head of the caudate nucleus was isolated and initially characterized by Schwartz et al. (2003); no gross or microscopic abnormalities were observed at this time.

Ethics Statement: Written informed consent for the donation of neonatal brain SVZ tissues was obtained from parents prior to tissue acquisition, under the auspices of the protocol for the National Human Neural Stem Cell Resource (NHNSCR) approved by the Children's Hospital of Orange County Institutional Review Board. All tissues were acquired in compliance with NIH and institutional guidelines.

GBM precursors were derived from 7 tumors taken at surgery (see Table 1); initial pathological review was followed by secondary neuropathological review to reconfirm diagnosis. Ethics Statement: Written informed consent for the donation of adult tumor brain tissues was obtained from patients prior to tissue acquisition, under the auspices of the protocol for the acquisition of human brain tissues obtained from the Ethical Committee board of the University of Padova and Padova Academic Hospital. All tissues were acquired following the tenets of the Declaration of Helsinki.

We dissociated [35] and cultured cells on fibronectin-coated dishes in DMEM/F12 (Irvine Scientific, Irvine, CA) supplemented with BIT9500 (1% bovine serum albumin, 10 µg/ml insulin, 200 µg/ml transferrin; Stem Cell Technologies, CA), 20 ng/ml basic fibroblast growth factor, (bFGF) and 20 ng/ml epidermal growth factor (EGF, both human and from R&D Systems, Minneapolis, MN) as previously described [36]. In vitro works are generally not fully representative of a reliable physiological condition. Although, a way to mimic tumor cell microenvironment is providing a lowered oxygen tension, which is characteristic of the solid tumor mass [9]; for this reason we cultured cells in an atmosphere of 2%, 5% carbon dioxide and the balance nitrogen, as previously described [36], [37]. Analyses of some metabolic parameters (i.e. lactate release and cytochrome c oxidase (COX) activity) indicate that, despite the use of high glucose concentration in our cell culture conditions (25 mM glucose), metabolic differences between tumor and normal cells are clearly detectable (Figure S2B,C). For continuous expansion, one-half of the medium was replaced every day and cultures were passaged every 7–10 days using TrypLE (Invitrogen, Carlsbad, CA). Cells were not cultured for more than 8 passages in vitro in order to avoid long term culture related effects. In some experiments, cells were supplemented with bone morphogenetic protein 2 (BMP2, 10 ng/ml or 50 ng/ml, R&D Systems) or rapamycin (100 µM, Sigma). Z-LLF-CHO (Z-LLF, Calbiochem, San Diego, CA), proteasome inhibitor, was used at the concentration of 30 µM added 30 min prior to BMP2 treatment. Esterificated diethyl-succinate (5 mM, Sigma) was added either alone or in combination with BMP2 (10 ng/ml) for 72 hr. To stabilize HIF-1α cells have been pre- treated for 12 hr with cobalt chloride (CoCl_2_, 100 µM, Sigma) prior to starting progressive high oxygen exposure and/or time course treatment with BMP2 the day after.

### Western blot and densitometric analysis

Total protein extracts were isolated in lysis buffer as described [37]. Equal amounts of protein (10–20 µg) were resolved using a SDS-PAGE gels and transferred to PVDF Hybond-p membrane (GE Healthcare), along with purified protein standard (Full Range Rainbow™ Molecular Weight Markers, from 10 to 250 kDa, GE Healthcare) to insure qualitative accuracy of analyzed proteins. Membranes were blocked with ECL Advance Blocking (Amersham Pharmacia, 2%) or I-block™ Blocking (Tropix, Sigma) for at least 1 hour or overnight, under rotation at RT or 4°C respectively. Membranes were then incubated overnight at 4°C under constant shaking with the following primary antibodies: Akt (rabbit, 1∶1000, Cell Signaling), Akt (T308) (rabbit, 1∶1000, Cell Signaling), HIF-1α (mouse, 1∶250, BD), mTOR (rabbit, 1∶1000, Cell Signaling), mTOR (S2448) (rabbit, 1∶1000, Cell Signaling), p70S6k (rabbit, 1∶1000, Cell Signaling), p70S6K (T389) (rabbit, 1∶1000, Cell Signaling), Stat3 (rabbit, 1∶1000, Cell Signaling), Stat3 (S727) (rabbit, 1∶1000, Cell Signaling), PHD2 (goat, 1∶300, Santa Cruz), REDD1 (rabbit, 1∶1000, Cell Signaling), FKBP38 (rabbit, 1∶200, Acris Antibodies GmbH) and β-actin (mouse, 1∶10000, Sigma). Membranes were next incubated with peroxidase-labeled goat anti-rabbit IgG (Sigma, 1∶100.000), peroxidase-labeled goat anti-murine IgG (Sigma, 1∶100.000) or donkey anti-goat IgG (Santa Cruz, 1∶150.000) for 60 min. All membranes were visualized using ECL Advance (GE Healthcare) and exposed to Hyperfilm MP (GE Healthcare). Densitometric analysis of the films was performed using Image J densitometer software. Values were normalized to β-actin and calibrated to control at 2% oxygen.

### Real-Time PCR analysis

RNA was isolated from cells using Trizol (Invitrogen) and 1 µg of total RNA reverse-transcribed using SuperScript RNAse H-Reverse Transcriptase (Invitrogen). Quantitative RT-PCR reactions were run in duplicate using Brilliant® SYBR® Green QPCR Core Reagent Kit (Stratagene, La Jolla, CA). Fluorescent emission was recorded in real-time (Sequence Detection System 7900HT, Applied Biosystems, Foster City, CA, USA). Gene expression profiling was completed using the comparative Ct method of relative quantification. Relative RNA quantities were normalized to beta-glucuronidase (GUSB) as a control and 2% oxygen, untreated group, was used as the calibrating condition. PCR amplification conditions consisted of 35 cycles with primers annealing at 56°C. The specificity of primers (see Table 2) was confirmed for every PCR run by dissociation curve analysis.

**Table 2:** Primer sets used for real-time PCR analysis.

Gene	Primer Sequence	Frag. Size
*PHD2*	F: 5′-GGGACATTCATTGCCTCACT-3′	158 bp
	R: 5′-ACACATGTGGTGCTTGCTGT-3′	
*GUSB*	F: 5′-GAAAATACGTGGTTGGAGAGCTCATT-3′	101 bp
	R: 5′-CCGAGTGAAGATCCCCTTTTTA-3′	
*FKBP38*	F: 5′-GGCTGTTGAGGAAGAAGACG-3′	187 bp
	R: 5′-TCCATGAGTGGGACACTGAG-3′	

Sample preparation and amplification are described in Materials and Methods. Abbreviations: F, forward primer; R, reverse primer; frag. size, fragment size; bp, base pairs.

### HRE-luciferase reporter assay

GBM cells and normal SVZ-derived cells were transfected using a Promega Corporation (Madison WI) protocol for transient transfection of adherent cells using Effectene reagent. HRE-luciferase reporter construct used (wHRE) was kindly provided by Dr. Indraccolo. It consists of a trimerized 24-mer containing 18 bp of sequence from the PGK promoter including the HRE (5′-tgtcacgtcctgcacgactctagt, HRE) and an 8-bp linker sequence followed by a 50-bp minimal tyrosine kinase promoter in a pGL2-firefly luciferase basic Vector backbone (Promega) [38]. The mutant HRE (mHRE) construct used to evaluate aspecific effects, has the ACG of the HIF-1 binding site mutated to CAT, abolishing binding, as well as a point mutation that abolished a BsgI restriction site for diagnostic purposes. GBM cells and normal SVZ-derived cells were transfected either with a wHRE or with a mHRE. Along with these vectors, also a Renilla luciferase vector has been transfected in order to normalize luciferase detection (Promega). 12 hr after transfection, total medium change was done and cells were treated with 50 ng/ml BMP2 for 7 hrs. Finally, cells were processed and analysis of HRE-luciferase activity was performed as described (Dual-Luciferase Reporter Assay System, Promega) by using a plate-reading luminometer (Victor, Perkin Elmer).

### Transduction of GBM cells using lentiviral vectors

The lentiviral plasmids containing HIF-1α siRNA and LUC siRNA target sequences, termed pLSLG-HIF-1α-siRNA and pLSLG-Luciferase-siRNA, respectively, were a kind gift of Dr. O.V. Razorenova (Department of Molecular Cardiology, Lerner Research Institute, Cleveland, OH) [39]. The lentiviral vectors were produced as previously described [40] and used to infect GBM cells and normal SVZ-derived cells. Target cells were incubated with stocks for at least 12 hr. Transduced cells were cultured for 5 days and analyzed by western blot.

### Succinate dehydrogenase activity

To evaluate aerobic metabolic activity succinate dehydrogenase (SDH) activity has been measured, since enzyme activity is considered an indicator of tricarboxylic acid (TCA) cycle activity. To measure SDH activity, we used the methods described by Levine et al. with little modifications [41]. Briefly, frozen cell monolayers (not fixed) were incubated for 20 min in the dark at 37°C in a reaction buffer containing 12.3 mM succinate (Sigma), 0.2 mM 1-methoxy-5-methylphenazine methyl sulphate (Sigma), 1.2 mM nitro blue tetrazolium (NBT, Sigma) in 50 mM Tris-HCl, pH 7.6. We used 12.3 mM malonate (Sigma) to test aspecific reactions, and we also tested 3-nitropropionic acid (3-NPA) (500 µM, Sigma), known to irreversibly inactivate SDH, as negative control. The enzyme activity is determined by measuring the formation of nitro blue diformazan (NBT-dfz) due to NBT reduction and the end product (i.e. blue cells) is visible by bright field microscopy (Olympus IX50) or can be measured at 570 nm.

### Lactate Assay

Extracellular lactate measurement was performed as directed (BioVision) using a lactate assay kit kindly provided by Prof. Burlina (Department of Paediatrics, University of Padova).

### COX activity analysis

Cell pellets were resuspended in PBS and lysed by two freeze/thaw cycles. COX activity was measured by the method of Salviati and co-workers [42]. Bovine cytochrome c was reduced with ascorbate and then desalted using a Sephadex G-25 column (Amersham). The concentration of the reduced substrate was determined spectrophotometrically at a wavelength of 550 nm. Spectrophotometrical measurements were performed using a Cary UV 100 spectrophotometer (Varian Inc., Walnut Creek, CA, U.S.A.).

### Statistical analysis

Graphs and statistical analyses were prepared using Prism 3.03 (Graph Pad). All values are presented as mean±standard error of the mean (S.E.M.). Statistical significance was measured by one-way ANOVA with Newman-Keuls multiple comparison post Test, *p<0.05, **p<0.01, ***p<0.001. For all graphs, an asterisk directly above a bar indicates a significant difference with its 2% oxygen counterpart; an asterisk over a bracket indicates a significant difference with another variable as indicated.

## Supporting Information

Figure S1 (A) Representative western blot analyses of indicated proteins; normal SVZ cells, initially expanded in 2% oxygen were treated as described in Fig. 4 for GBM cells. (B) Representative western blot analyses of indicated proteins extracted from normal SVZ cells that have been treated for 12 hr with CoCl2 (100 µM, Sigma) either at 2% oxygen or 20% oxygen, starting progressive time course treatment with BMP2 the day after. (C-H, K) Bar graphs showing mean intensity of indicated proteins normalized to control at 2% oxygen (corresponding to the 0 base line)±S.E.M. comparing 3 different normal SVZ cultures, n = 3 for each one. Statistical analyses were done comparing each time point at 2% O2 to its respective time point at 20% O2. (I, J) QRT-PCR analyses of PHD2 and FKBP38 normalized to GUSB and then calibrated to 2% oxygen control (ΔΔCt Method), mean±S.E.M. comparing 2 different normal SVZ cultures, n = 3 for each one.
(1.08 MB TIF)

Figure S2 (A) Representative citochemical analysis of SDH activity by using NBT reduction methodology in normal SVZ cells, treated as described in Fig. 8A. (B) Bar graph showing extracellular lactate measure comparing 3 different GBM cell cultures and 2 different normal SVZ cell cultures. (C) Bar graph showing cytochrome c oxidase (COX) activity comparing 3 different GBM cell cultures and 2 different normal SVZ cell cultures. Cells have been either maintained under hypoxia or exposed to acute high oxygen tension for 48 hr.
(4.37 MB TIF)
