# Supplementary figures and images for: Molecular Mechanisms of HIF-1α Modulation Induced by Oxygen Tension and BMP2 in Glioblastoma Derived Cells
Source: PLoS One. 2009 Jul 9;4(7):e6206. doi: 10.1371/journal.pone.0006206 (PMC2702690; doi:10.1371/journal.pone.0006206)

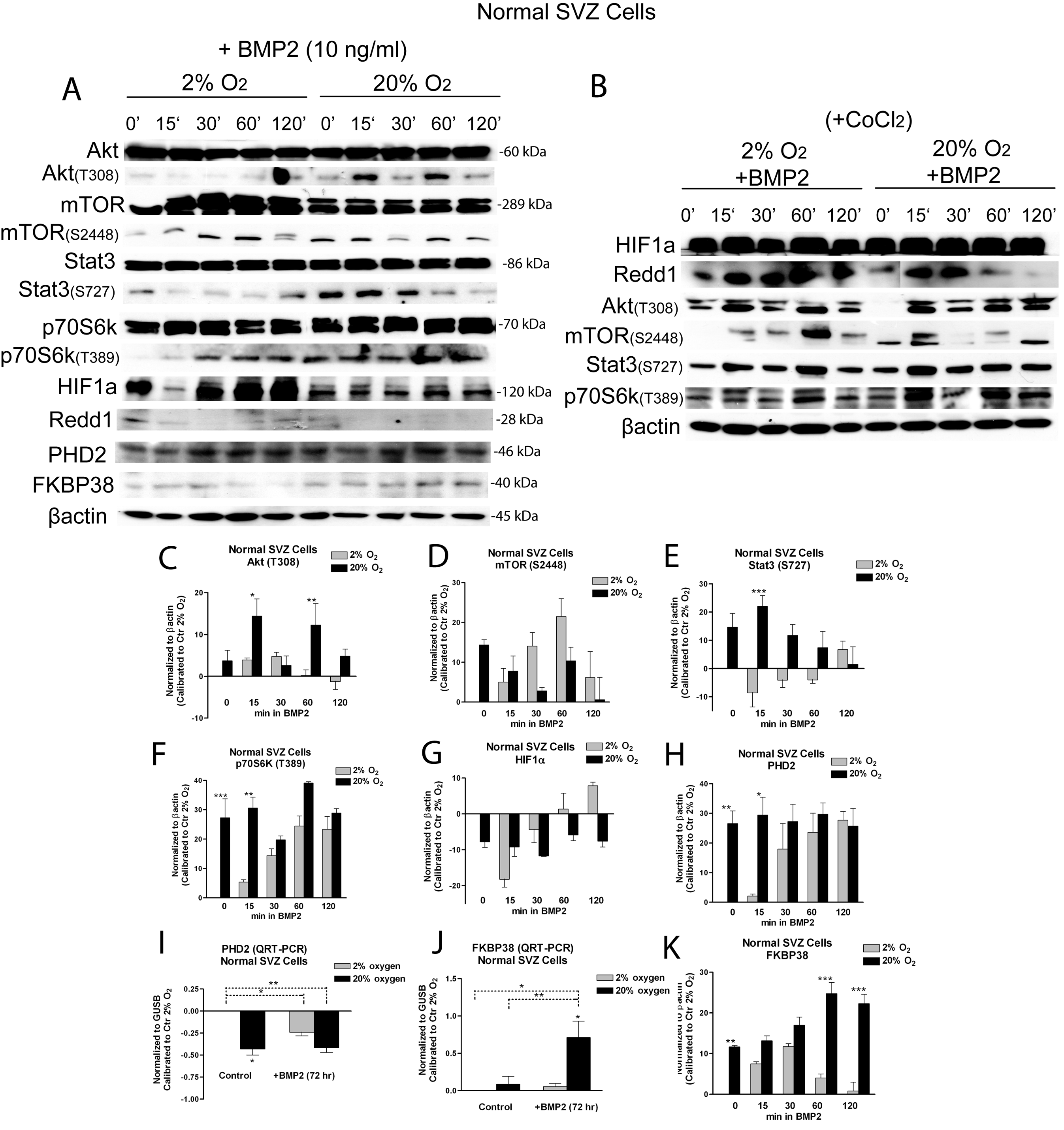

Supplement: Figure S1 — (A) Representative western blot analyses of indicated proteins; normal SVZ cells, initially expanded in 2% oxygen were treated as described in Fig. 4 for GBM cells. (B) Representative western blot analyses of indicated proteins extracted from normal SVZ cells that have been treated for 12 hr with CoCl2 (100 µM, Sigma) either at 2% oxygen or 20% oxygen, starting progressive time course treatment with BMP2 the day after. (C-H, K) Bar graphs showing mean intensity of indicated proteins normalized to control at 2% oxygen (corresponding to the 0 base line)±S.E.M. comparing 3 different normal SVZ cultures, n = 3 for each one. Statistical analyses were done comparing each time point at 2% O2 to its respective time point at 20% O2. (I, J) QRT-PCR analyses of PHD2 and FKBP38 normalized to GUSB and then calibrated to 2% oxygen control (ΔΔCt Method), mean±S.E.M. comparing 2 different normal SVZ cultures, n = 3 for each one. (1.08 MB TIF) [file pone.0006206.s001.tif]

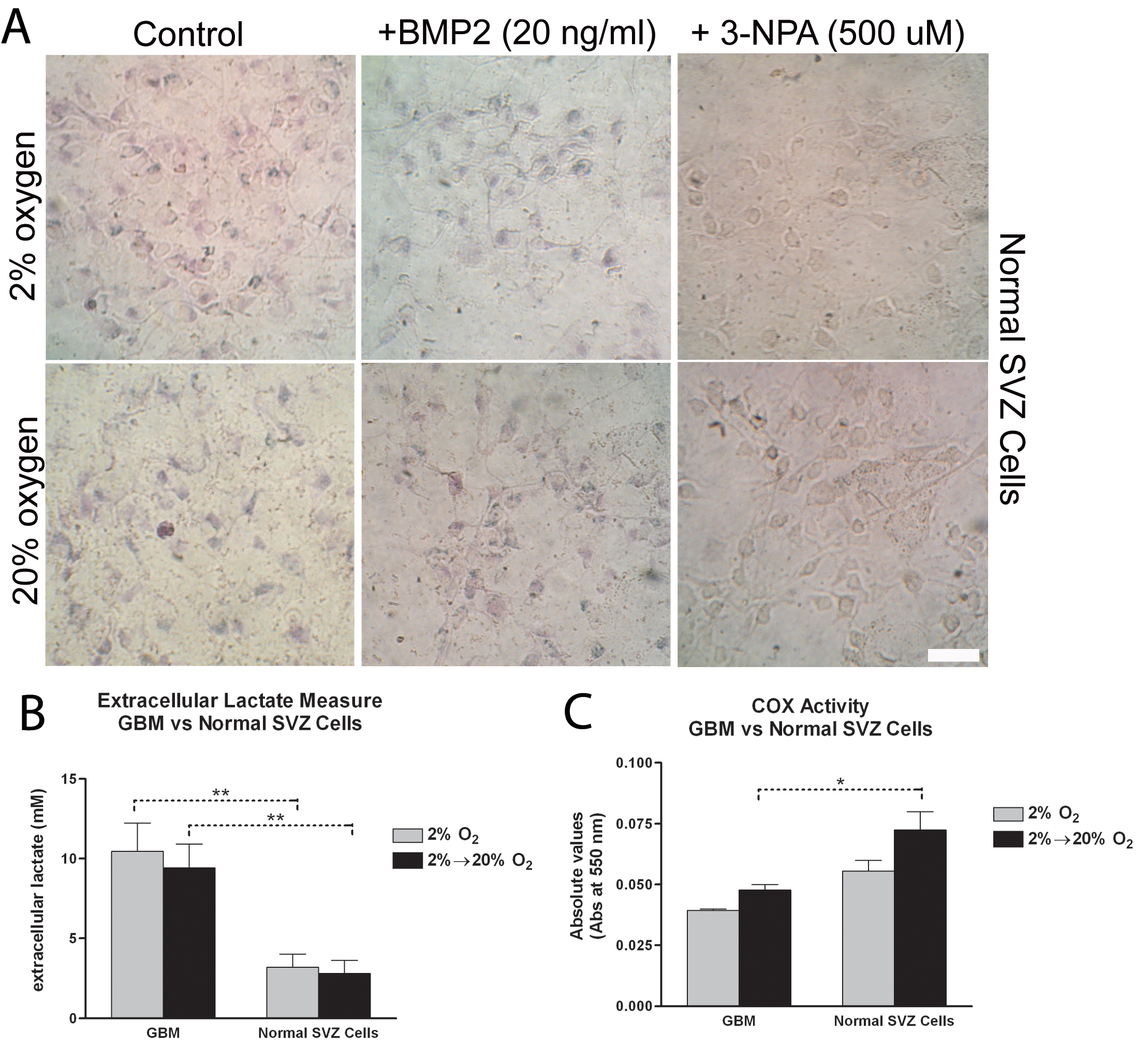

Supplement: Figure S2 — (A) Representative citochemical analysis of SDH activity by using NBT reduction methodology in normal SVZ cells, treated as described in Fig. 8A. (B) Bar graph showing extracellular lactate measure comparing 3 different GBM cell cultures and 2 different normal SVZ cell cultures. (C) Bar graph showing cytochrome c oxidase (COX) activity comparing 3 different GBM cell cultures and 2 different normal SVZ cell cultures. Cells have been either maintained under hypoxia or exposed to acute high oxygen tension for 48 hr. (4.37 MB TIF) [file pone.0006206.s002.tif]
